# Patient perspectives on treatment-related toxicities and therapeutic drug monitoring with tyrosine kinase inhibitors for the treatment of non-small-cell lung cancer

**DOI:** 10.1177/17588359241303403

**Published:** 2024-12-07

**Authors:** Judith Gulikers, Jeroen Bruinsma, Janna Schoenmaekers, Safiye Dursun, Vivianne Tjan-Heijnen, Robin van Geel, Sander Croes, Lizza Hendriks

**Affiliations:** Department of Clinical Pharmacy & Toxicology, Maastricht University Medical Centre+, Maastricht, The Netherlands; CARIM School for Cardiovascular Disease, Maastricht University, Maastricht, The Netherlands; Department of Health Promotion, CAPHRI – Care and Public Health Research Institute, Maastricht University, Maastricht, The Netherlands; Department of Pulmonary Diseases, GROW – School for Oncology and Reproduction, Maastricht University Medical Centre+, Maastricht, The Netherlands; Department of Pulmonary Diseases, GROW – School for Oncology and Reproduction, Maastricht University Medical Centre+, Maastricht, The Netherlands; Division Medical Oncology, GROW – School for Oncology and Reproduction, Maastricht University Medical Centre+, Maastricht, The Netherlands; Department of Clinical Pharmacy & Toxicology, Maastricht University Medical Centre+, Maastricht, The Netherlands; CARIM School for Cardiovascular Disease, Maastricht University, Maastricht, The Netherlands; Department of Clinical Pharmacy & Toxicology, Maastricht University Medical Centre+, Maastricht, The Netherlands; CARIM School for Cardiovascular Disease, Maastricht University, Maastricht, The Netherlands; Department of Pulmonary Diseases, GROW – School for Oncology and Reproduction, Maastricht University Medical Centre+, PO Box 5800, Maastricht 6202 AZ, The Netherlands

**Keywords:** non-small-cell lung cancer, patient perspectives, therapeutic drug monitoring, toxicities, tyrosine kinase inhibitor

## Abstract

**Background:**

Tyrosine kinase inhibitors (TKIs) have significantly improved treatment-related outcomes of patients with oncogene-driven non-small-cell lung cancer (NSCLC). TKIs are usually well tolerated and used for a prolonged time, although experienced toxicity varies between patients. It is unclear whether patients report all (low grade) toxicities and how these impact their daily lives. The use of therapeutic drug monitoring (TDM) to, for example, manage toxicities is increasingly applied, but there is limited insight into the patient perspective regarding TDM. This qualitative study aims to explore patient perspectives on TKI toxicity and TDM.

**Methods:**

Five semi-structured focus group interviews were held, each with three to four patients with NSCLC using a TKI and their (care) partners. Two researchers independently performed a directive content analysis.

**Results:**

In total, 16 patients and 12 (care) partners participated. Experienced treatment-related toxicities were encountered limitedly and patients felt no boundaries discussing these with their treatment team. However, symptoms were sometimes not reported as they were doubted as treatment related. The concept of TDM-guided dosing to, for example, reduce TKI exposure to account for dosing outside the therapeutic window resulted in feelings of uncertainty regarding treatment efficacy. Patients emphasized the need for thorough research and frequent check-ups to ensure treatment efficacy.

**Conclusion:**

Perceived TKI-related toxicities seem limited, although the treatment team should pay attention to symptoms not directly described by patients as treatment related. In general, patients are open to implement TDM-guided dosing, but only if thorough scientific evidence demonstrates retained or enhanced safety and efficacy.

## Plain language summary

Patient perspectives on side effects and therapeutic drug monitoring with tyrosine kinase inhibitors for the treatment of non-small cell lung cancer

Background: Tyrosine kinase inhibitors (TKIs) have greatly improved the outcomes for patients with non-small cell lung cancer (NSCLC). These drugs are generally well tolerated and used for long periods, although the experienced side effects can vary between patients. It is not clear if patients always report mild side effects and how these affect their daily lives. Therapeutic drug monitoring (TDM), which is used to adjust drug doses to manage side effects, is becoming more common, but the patient’s perspective on this is largely unknown. This study explores patient perspectives on potential side effects of TKIs and the use of TDM. Methods: Five group interviews with 3 to 4 patients each, who were taking TKIs, and their caregivers or partners were held. Two researchers analyzed the data independently. Results: A total of 16 patients and 12 caregivers participated. Most patients experienced few side effects and felt comfortable discussing these with their healthcare team. However, some symptoms were not reported because patients were unsure if they were related to the treatment. The idea of using TDM to adjust doses caused some patients to feel uncertain about whether their treatment would still work effectively. They stressed the importance of thorough research and regular check-ups to ensure the treatment remains effective. Conclusion: While patients generally experience few side effects from TKIs, healthcare teams should be attentive to symptoms patients may not directly associate with the treatment. Overall, patients are open to using TDM to adjust their doses, but only if there is strong scientific evidence proving it is safe and effective.

## Background

Oral tyrosine kinase inhibitors (TKIs) are frequently used in patients with non-small-cell lung cancer (NSCLC) harbouring actionable genomic alterations as they provide significantly improved outcomes (survival as well as the quality of life).^1,2^ These drugs are generally used for a prolonged period of time and are usually well tolerated, although the experienced toxicities vary between patients. Even though low-grade toxicities can still be burdensome and negatively affect the quality of life of patients, they are usually not highlighted in clinical trial publications and reports.^2,3^ A study investigating skin-related adverse events in patients with NSCLC receiving TKIs reported that these adverse events had a negative impact on the quality of life of these patients but also reported that this was strongly influenced by age, disease stage and comorbidities.^4^ The overall patient perspective regarding experienced treatment-related toxicities was not explored while the discussion of (low grade) toxicity is very important to provide strategies to mitigate their impact on quality of life (QoL). Previous investigations in patients receiving chemotherapy, immunotherapy and a limited number of patients receiving TKIs reported discrepancies between toxicities reported by physicians, nurses and patients. It was noticed that not all experienced toxicities reported by patients were also reported by nurses and even less by treating physicians.^5–8^ This may be caused by, for example, differences in the interpretation of treatment-related toxicities or difficulties in the physician–patient relationship. It is possible that physicians only report toxicities related to the treatment, whereas the nurse and patient report all symptoms, not necessarily related to the treatment. Furthermore, patients may not share their experienced side effects for fear of risk of decreased survival upon a subsequent decision for dose reduction or treatment discontinuation. Another reason could be that patients perceive low-grade toxicities as part of the treatment that cannot be avoided, despite that for some adverse effects, adequate measures can be taken to reduce their impact or severity.^9,10^ Whether this also applies to patients receiving a TKI for NSCLC is currently unknown.

Additionally, in the current treatment landscape, personalizing targeted therapies via therapeutic drug monitoring (TDM) is increasingly studied. TDM-guided dosing may help to prevent ineffective treatment caused by sub-therapeutic drug concentrations, or conversely, unnecessarily high drug exposure potentially resulting in an increased risk of toxicity.^11,12^ TDM-guided dosing is already part of standard practice for some antibiotics and is also applied for some TKIs, such as sunitinib, pazopanib and imatinib.^13–15^ However, as patient participation is essential to study further implementation and explore the added value of TDM-guided dosing in NSCLC, it is important to take the patient’s perspective into account.

Therefore, we conducted this qualitative study to explore the perspective of patients and the view of their (care) partner regarding TKI toxicities and the reporting of adverse effects, including barriers to reporting, and the coping mechanisms to deal with these toxicities. Additionally, we explored their views on the use and incorporation of TDM during TKI treatment.

## Methods

Five focus groups were conducted in September and October 2023 at the Maastricht University Medical Centre+ (MUMC+), the Netherlands. Each group consisted of three to four patients with, if possible, their (care) partner (either partner or a deeply involved friend). These focus groups allowed for an in-depth understanding of the experiences of patients regarding TKI toxicity throughout the disease trajectory and allowed us to explore their perspective regarding TDM of TKIs. The findings are reported according to the consolidated criteria for reporting qualitative research.^16^

### Participants

Eligible patients and their (care) partners were recruited and selected from the thoracic oncology outpatient clinic of the comprehensive cancer centre at MUMC+. Instead of the (care) partner, a deeply involved friend of the patient was also allowed to participate if they were important for the patient’s well-being. Separate informed consent documents were available for patients and their (care) partners.

They were considered eligible if their age was 18 years or older, and for patients, if they received a TKI for the treatment of NSCLC for at least 6 months prior to inclusion. A minimum TKI use of 6 months was chosen to ensure adequate time for the development of potential treatment-related toxicities. Patients and partners who were not able to understand the study information or informed consent procedure, or those who were not able or willing to participate in a group discussion were excluded from the study.

### Focus groups

The focus groups lasted around 90 min each and were moderated by two investigators (J.B. and J.G.). One investigator (J.B.: PhD in health sciences, male) had prior experience with moderating focus groups and led the discussion. The second investigator (J.G.: a PhD candidate in clinical pharmacology and toxicology, female) kept track of the pre-defined themes that were discussed during the session and provided additional information upon request from participants. It was possible that the participants knew one of the investigators from participation in previous research; however, the investigators were not part of the patient’s treatment team. For all participants, each focus group started by filling out a short questionnaire with sociodemographic information. In case data saturation was not achieved after the fifth pre-planned focus group, additional sessions could be held.

The pre-defined themes discussed during the session included: (a) perceived toxicities and coping, (b) the therapeutic relationship with the thoracic oncologist(s) and oncology nurse(s) including the reporting of toxicities, (c) (care) partners’ experience regarding toxicities and coping mechanisms, (d) used sources of information and peer support and (e) perspectives regarding future implementation of TDM-guided dosing of the TKI. An English translation of the questions discussed during the sessions is shown in Supplemental Appendix I. A patient representative was involved in designing the final questions for the focus groups.

### Analysis

The focus groups were audio-recorded and transcribed verbatim while pseudo-anonymizing the personal information of participants. Using a directive content analysis, the transcripts were open coded in ATLAS.ti software (version 23) independently by the two investigators who also moderated the focus groups.^17^ After the independent analysis of each transcript, discrepancies in coding were listed and discussed to reach a consensus about the codes and perform axial coding. Subsequently, (sub)categories were identified through discussion and visualized in a mind map to facilitate an in-depth understanding of the codes and (sub)themes. The main findings were refined through discussion within the research team including a health scientist, experts in thoracic oncology and a representative of a patient organization.

### Ethical consideration

This study was approved by the Medical Ethics Committee of MUMC+ (METC 2023-3719) and conducted according to the declaration of Helsinki. All participants provided written informed consent prior to the start of the focus group.

## Results

### Participants

**Figure 1.**
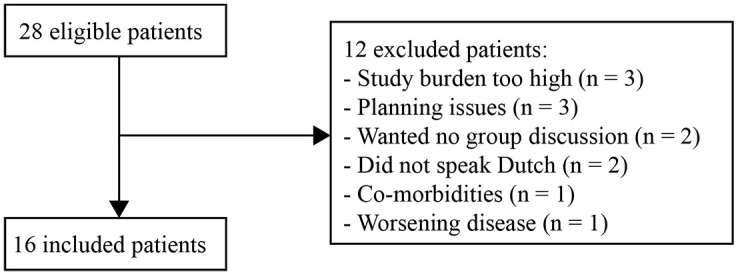
Flowchart of included patients.

A total of 16 patients and 12 (care) partners participated in five focus groups. In the recruitment phase, 12 other patients were approached but refrained because they perceived the study burden as too high (*n* = 3), experienced planning issues (*n* = 3), did not want to participate in a group discussion (*n* = 2), did not speak Dutch (*n* = 2), had comorbidities interfering with participation (*n* = 1) or experienced sudden worsening of NSCLC (*n* = 1) (Figure 1). Demographics are shown in Table 1. The median age of the patients was 64.5 years (range 42–80) and of the (care) partners 65.0 years (range 58–78). Alectinib was the most used TKI (56.3%) and the median time on the currently used TKI was 44.7 months (range 10.6–104.9). Most patients received the TKI in the first or second line of treatment (81.3%). Most (care) partners were married to the patient (91.7%).

**Table 1.** Baseline characteristics of patients and (care) partners.

Baseline characteristics	Patients (*n* = 16)	(Care) Partners (*n* = 12)
Age, years (range)	64.5 (42–80)	65 (58–78)
Sex, *n* (%) (women)	10 (62.5)	6 (50)
Marital status, *n* (%)		
Living together	0 (0)	NA
Married	14 (87.5)	NA
Single	2 (12.5)	NA
Relation to patient, *n* (%)		
Life partner	NA	11 (91.7)
Care partner/deeply involved friend	NA	1 (8.3)
Highest education received, *n* (%)		
Primary education	0 (0)	0 (0)
Secondary education	6 (37.5)	2 (1.7)
College	6 (37.5)	6 (50)
University of applied sciences	4 (25)	3 (25)
University	0 (0)	1 (8.3)
Line of treatment, *n* (%)		
1	8 (50)	NA
2	4 (25)	NA
3	2 (12.5)	NA
4	2 (12.5)	NA
TKI, *n* (%)		
Alectinib	9 (56.3)	NA
Dabrafenib + Trametinib	2 (12.5)	NA
Osimertinib	2 (12.5)	NA
Erlotinib	1 (6.3)	NA
Lorlatinib	1 (6.3)	NA
Crizotinib	1 (6.3)	NA
Time on TKI, months (range)	44.7 (10.6–104.9)	NA
Dose reduction due to toxicities^a^, *n* (%)	5 (31.3)	NA

a Dose reductions received at current or previously received TKI.

NA, not applicable; TKI, tyrosine kinase inhibitor.

### Analysis of the focus groups

Directive content analysis resulted in the identification of five major themes: treatment-related toxicities, consultation of and contact with the treatment team, information and peer support, partners’ experience, and TDM-guided dosing of TKIs. A list of used codes is shown in Supplemental Appendix II.

#### Treatment-related toxicities

A wide range of potential treatment-related toxicities was discussed by the participants. The most frequently mentioned toxicities were skin related, including sun sensitivity, dry skin, rashes and pruritus. Additionally, patients reported changes in the structure of their hair and nails. Other common complaints involved fatigue, muscle cramps, muscle pain and gastrointestinal issues.

Most patients found it challenging to pinpoint the exact source of the experienced toxicity. For example, they openly questioned whether these adverse effects were due to the TKI, other medications, previous treatment, the tumour itself or even ageing. Additionally, some patients recognized certain toxicities only after hearing others describe certain complaints:

*I have some complaints, such as nail problems, but then I wonder if they are the result of the anti-cancer medication or if they started before*. – Male, 65 years old, osimertinib user

*Many things you don’t recognize as toxicity until somebody else mentions them*. – Male, age 70 years old, partner alectinib user

Overall, patients were satisfied and glad to receive an oral TKI instead of other (parenteral) cancer treatments, such as chemotherapy. Frequently, participants described they belonged to the lucky few and felt that the use of TKIs was a game changer as adverse effects were relatively mild and the tumour remained stable, often for several years. In return, they accepted the perceived toxicities as part of the treatment and tried to avoid dose reductions because they feared the TKI would be less effective.

*There are some side effects, but I will not complain since I am up and about*. – Male, 62 years old, erlotinib user

*We did discuss it [TKI dose reduction], but I would rather have swollen hands and feet. Please don’t change the medication as my cancer is currently controlled*. – Female, 58 years old, crizotinib user

#### Consultation and contact treatment team

Patients often did not feel the urgency to contact their treatment team immediately upon experiencing mild treatment-related toxicities and tried to deal with the toxicity by themselves first. Of note, most did not perceive any barriers to discuss toxicities with the treatment team, but occasionally refrained from doing so due to doubts about whether the TKI was the cause:

*She did not immediately sound the alarm when she had side effects. She first tried to cope with it by herself*. – Female, 63 years old, partner dabrafenib and trametinib user

*You expect toxicities, which are unpleasant but most of them are manageable. However, there is a point when it becomes overwhelming. For instance, as soon as you continuously vomit, are unable to eat, and begin to dehydrate. That is the time to contact your physician*. – Male, 73 years old, partner dabrafenib and trametinib user

*If I have certain symptoms then I would think it is part of aging and then I would not discuss it with my physician. So it’s not the physician’s fault, it’s more my own fault that I do not discuss it myself*. – Female, 75 years old, alectinib user

Some of the participating patients and (care) partners were critical of their physician not being proactive. They argued that they had to be proactive themselves to take measures to reduce toxicity or to receive the scans or follow-up appointments as they requested. Notably, other patients and (care) partners did not recognize this problem. It seems that a previous history of a missed diagnosis or progression under treatment highly influenced these feelings:

*You have to be annoying in a polite way, otherwise you will end up at the back of the line*. – Male, 62 years old, erlotinib user

*When I told the physician the side effects worsened, she asked if she could be of any help. She actually listens and takes all necessary time during our appointments*. – Female, 64 years old, partner lorlatinib user

Patients and partners discussed that at the start of the treatment, they would occasionally visit a nurse before consultation with the physician. Although this visit was generally described as fine, some patients did not see the added value. They argued that medical questions were not adequately answered and that they would rather talk to the physician:

*Initially, I did think seeing a nurse would be interesting and that they would be easy to approach, maybe easier than the oncologist. But when I asked her questions, she [the nurse] would tell me that these were medical questions and that I had to ask the physician*. – Male, 65 years old, osimertinib user

#### Information and peer support

Patients perceived the treatment team as a primary and reliable source of information about the TKI and treatment-related toxicities. For additional information, patients referred to the information leaflet they received at the start of the TKI treatment, which included information on self-management of low-grade toxicities, or indicated to search for additional information online. When searching online, some patients used Dutch or international Facebook groups. These groups were mainly used to stay up to date with scientific developments, to find peers who experience similar symptoms or to find those who had a similar lung cancer diagnosis to compare treatment options. Sharing personal experiences through social media platforms was perceived as less useful:

*I did receive an information leaflet during my first visit which contained all sorts of information, but I just know that I have NSCLC and I am not that fond of Google. So, I will just let it happen and will see what is coming*. – Female, 72 years old, alectinib user

*I am part of the [Facebook] group for scientific reasons, just to be up to date with new treatments becoming available and ongoing studies. When I read posts of others who are in doubt, I occasionally wonder whether I should block it or leave the group. However, the scientific input and developments are the reason why I am still part of this group*. – Female, 42 years old, alectinib user

Regarding peer support, most patients only had occasional contact with other patients, for example when participating in scientific research. The few patients who were in contact with peers with a similar diagnosis, either in real life or online, were satisfied with this contact and felt it resulted in recognition and support. Some patients were not open towards peer support and perceived it as too confronting, were not interested or did not have the time:

*I am part of a forum with others who use osimertinib and then you do read about similar adverse effects. That does give some recognition*. – Female, 56 years old, osimertinib user

*It [contact with peers] was all negative and mainly people who were afraid of dying. I swore to myself that I would not go there again. It did not do me well*. – Female, 70 years old, dabrafenib and trametinib user

#### Partners’ experience

Partners who participated during the focus group sessions were in general closely involved in the treatment of the patient and the communication between the patient and his or her (care) partner was often described as good. Treatment-related toxicities were often also seen by the (care) partner, but the patient was mostly the one who discussed these with the treatment team. Some partners described noticing cognitive changes while the patient was unaware of them and mentioned they discussed this during outpatient visits:

*We talk about the side effects and she [the patient] also points it out*. – Male, 63 years old, partner alectinib user

*He does not notice or bother with the side effects, but I do*. – Female, 64 years old, partner lorlatinib user

#### TDM-guided dosing of TKIs

Patients and (care) partners were asked to share their ideas regarding the implementation of TDM-guided dosing of the TKI. Depending on the consequences of TDM, this was valued and patients were open to increase the TKI dose if this could improve the efficacy of the treatment. By contrast, the suggestion of dose reduction without having severe treatment-related toxicities mainly led to feelings of insecurity, uncertainty and unrest, even if plasma (trough) levels of the TKI were above average or even outside the therapeutic window. According to patients, the high efficacy of the treatment was their primary concern. A few patients described they wished to receive as much TKI as possible as they assumed a higher exposure would have more effect. On the other hand, some perceived it as important to receive a low but still optimal TKI dose which also maintains an adequate plasma (trough) level for optimal efficacy to lower the risk for toxicities:

*Reducing the TKI dose would make me feel insecure. The pills are currently effective and the treatment should not be stopped or reduced*. – Female, 56 years old, osimertinib user

*For me, there are two sides to this story. On the one hand is security. As long as I receive enough of the anti-cancer medication, then everything will go well. On the other hand, the less of the medication I take, the less side effects, whatever they are. So on one hand I would say as low as possible, but on the other hand as high as possible, just to be sure*. – Male, 69 years old, alectinib user

When asked about critical terms to implement TDM, patients mentioned that frequent scans and follow-up appointments were key to implement TDM-guided dosing of TKIs. Additionally, they expressed being open to it but only if efficacy of TDM-based dose optimisation has been proven before:

*I would be open for it [the implementation of TDM], but it should be proven [safe, without impact on antitumor efficacy]. It should not still be under investigation*. – Female, 75 years old, alectinib user

*I would find it [reduction of TKI dose] scary. If it has been investigated and it is proven effective, than I would find it very positive*. – Female, 58 years old, crizotinib user

## Discussion

This study explored relevant topics related to the perspectives of patients with NSCLC towards treatment-related toxicities of TKI treatment in NSCLC and the implementation of TDM-guided dosing. Patients enrolled in this study experienced a wide range of treatment-related toxicities that were in general perceived as tolerable. Patients did not feel boundaries in discussing toxicities with their treatment team. However, the primary reasons not to discuss the experienced adverse effects with their treatment team included patients perceiving these as unavoidable consequences of the treatment, not recognizing the toxicities as treatment-related adverse effects, and the low-grade toxicities were often self-manageable. Additionally, some patients preferred to speak directly with their physician rather than to a nurse. Information and peer support were mainly used to stay up to date with ongoing research and to learn more about new treatment options, that might become available in the short term. Overall, patients were hesitant towards the implementation of TDM-guided dosing of TKIs because they felt it may be less effective compared to traditional dosing. They stressed the importance of research to ensure maintaining or enhancing TKI efficacy of TDM-based dosing and highlighted the importance of frequent check-ups during the implementation of TDM.

The majority of the included patients consist of patients with *ALK*-positive NSCLC. In the Netherlands, patients with a rare oncogenic mutation can only be treated in an academic, designated centre for rare (defined as <5% incidence in NSCLC, e.g., *ALK*) driver mutations in NSCLC, while patients with a classical *EGFR* ex19del or *EGFR* ex21 L858R can be treated in all Dutch hospitals. This study was performed in an academic hospital. Therefore, the number of patients with *ALK*-positive NSCLC is higher than one would expect from a mixed NSCLC cohort. The proportion of patients with *EGFR*-mutated NSCLC was in line with expectations.

Skin-related toxicities were frequently mentioned by patients, but the impact of these treatment-related toxicities was limited. Du et al. and Tseng et al. investigated patients with skin-related toxicities from treatment with EGFR-TKIs and argued that this substantially impacted the QoL of these patients, which was not in line with our observations.^4,18^ The introduction of newer (third generation) EGFR-TKIs might be the clarification. Due to the higher selectivity against the mutated EGFR receptor, osimertinib is associated with less severe skin and gastrointestinal toxicity. Tseng et al., indeed, only included patients who received gefitinib, erlotinib or afatinib, which are known to lead to more severe toxicities than osimertinib.^19^ The study by Du et al. did not differentiate between TKIs used. Other factors such as high age, comorbidities and stage of the disease were also found to substantially impact QoL in the study of Du et al. and are expected to be more critical factors.

As described above, Cirillo et al. reported that the adverse effects reported by the nurse were more similar to the adverse effects reported by the patient than the ones reported by the physician.^7^ The authors already speculated that the differences between physicians and nurses might have occurred because the physician tends to only report toxicities that he/she feels are directly caused by the treatment and the nurse tends to describe all toxicities. Another possible reason for this discrepancy was that patients had a closer connection to the nurse with whom they would discuss adverse effects more easily. In our study, we observed the opposite as patients preferred to discuss their treatment (related toxicities) directly with the treating physician. This could be caused by cultural differences as the patients enrolled by Cirillo were Italian while we enrolled solely Dutch patients. Another difference was that most patients in the current study did not speak with a nurse regularly, in contrast with the patients included in the study by Cirillo et al.

Regarding TDM-guided dosing, patients and their (care) partners expressed feelings of uncertainty and unrest when discussing the possibility of receiving a lower dose of the TKI even if lowering the TKI dose would minimize treatment-related toxicities while maintaining efficacy against the tumour. Unrest with regard to the optimization of oncolytic drug use was also expressed by patients in the study by Van Rooijen-Schuurman et al., where general oncolytic care was discussed with patients and caregivers.^20^ They emphasized the role of the pharmacy, although this also entailed the evaluation of interactions with supplements (herbs or food additives) or other drugs. Additionally, patients in the current study were hesitant to participate in hypothetical research investigating the effect of TDM-guided dosing. An important consideration, however, is that the patients included in the current study were long-term TKI users, which could have influenced their willingness, not to risk the long-term achieved stability of their tumour. Additionally, results might have been different if the need for TDM-guided dosing in relation to financial, and therefore societal, impact was discussed. Improvement of cost-effective use of TKIs is essential to allow new TKIs to be approved and reimbursed and eventually to become available to patients. In contrast to our study, a study by Chen et al. on QoL and mental health in patients with chronic myeloid leukaemia (CML) reported that the majority of patients (~80%) were open to dose reduction of their TKI treatment for financial reasons, poor QoL due to treatment-related toxicities and worries about the side effect of long-term use.^21^ Important to note is that Chen et al. only included patients who were in the chronic, and therefore stable, phase of CML. This is an important difference when compared to the patients with advanced NSCLC included in our study. Currently, trials investigating TDM-guided dosing of for example alectinib are ongoing, although the scope of this study is to achieve a minimal alectinib plasma concentration and dose reductions based on high plasma levels are not part of the study design.^22^ Some retrospective studies have been performed on the effectiveness of low-dose TKI treatment and did show comparable effectivity compared to the standard dose, but most patients did have dose-limiting toxicities on the standard dose which led to the dose reduction.^23,24^

To evaluate the perspectives of patients and (care) partners, we decided to organize focus groups rather than asking participants to fill out a questionnaire. By organizing focus groups, we anticipated that patients and (care) partners would participate in an active discussion and that it would help to share experiences with treatment-related toxicities. This would not have been possible with standardized questionnaires. One limitation is, however, that the semi-unstructured nature of this study design does not allow for extensive statistical analysis and that the results obtained from the focus groups only indicated the general patient perspective. However, the obtained results allow for further investigation especially regarding TDM-guided dosing. It is, furthermore, important to note that patients participating in these focus groups might already discussed their diagnosis and experienced toxicities more easily than patients who refrained from participation in these group sessions. Some of the patients refused to participate but mentioned being open for a one-on-one interview. We applied minimal inclusion and exclusion criteria to achieve an adequate representation of the patients who receive a TKI in the thoracic oncology unit of our comprehensive cancer centre. This, however, may have resulted in a high number of patients who used alectinib because the alectinib users compile a substantial part of the long-term TKI users in our hospital. Furthermore, there were minimal cultural or ethnic differences between the participants, which could have influenced the results. We may have failed to include participants who experienced severe toxicities necessitating the discontinuation of a TKI because we only included patients who actively used TKIs for 6 months or more. We did include some patients (31.3%) who changed TKI upon progression during their previous line of treatment or received a dose reduction as a response to severe toxicities.

## Conclusion

In conclusion, experienced treatment-related toxicities were limited and could easily be discussed with the treatment team by the patients who participated in these focus groups. Nevertheless, the treatment team should pay attention to symptoms not directly described by the patients as treatment related. Increasing the TKI dose based on TDM was generally received well, while TKI dose reductions based on TDM were accompanied by the feeling of unrest and uncertainty. These concerns should be taken into account when designing studies investigating further implementation of TDM in NSCLC and when informing potentially eligible patients about these studies. Therefore, future implementation should be based on thorough scientific evidence demonstrating safety and efficacy, accompanied by adequate and regular monitoring.

## Supplemental Material

sj-docx-1-tam-10.1177_17588359241303403 – Supplemental material for Patient perspectives on treatment-related toxicities and therapeutic drug monitoring with tyrosine kinase inhibitors for the treatment of non-small-cell lung cancer Supplemental material, sj-docx-1-tam-10.1177_17588359241303403 for Patient perspectives on treatment-related toxicities and therapeutic drug monitoring with tyrosine kinase inhibitors for the treatment of non-small-cell lung cancer by Judith Gulikers, Jeroen Bruinsma, Janna Schoenmaekers, Safiye Dursun, Vivianne Tjan-Heijnen, Robin van Geel, Sander Croes and Lizza Hendriks in Therapeutic Advances in Medical Oncology

## Declarations

**Ethics approval and consent to participate:** This study was approved by the Medical Ethics Committee of MUMC+ (METC 2023-3719) and conducted according to the declaration of Helsinki. All participants provided written informed consent prior to the start of the focus group.

**Consent for publication:** All participants gave consent for publication via written informed consent prior to the start of the study.

**Availability of data and materials:** Data are available upon reasonable request.
