# Supplementary material for: Patient perspectives on treatment-related toxicities and therapeutic drug monitoring with tyrosine kinase inhibitors for the treatment of non-small-cell lung cancer
Source: Ther Adv Med Oncol. 2024 Dec 7;16:17588359241303403. doi: 10.1177/17588359241303403 (PMC11624549; doi:10.1177/17588359241303403)
Supplement: sj-docx-1-tam-10.1177_17588359241303403 – Supplemental material for Patient perspectives on treatment-related toxicities and therapeutic drug monitoring with tyrosine kinase inhibitors for the treatment of non-small-cell lung cancer [file sj-docx-1-tam-10.1177_17588359241303403.docx]

**Appendix I - Guiding questions focus groups**

- Introducing moderators and focus group participants
- General questions
  - Do you experience adverse effects from the tyrosine kinase inhibitor (TKI) or did you experience this in the past?
  - Do you discuss these adverse effects with your partner?
- Contact with treatment team
  - Do you discuss the experienced adverse effects with your treatment team (doctor or nurse)? Are there items you do not discuss with the doctor and do discuss with the nurse or *vice versa*?
  - Do you immediately discuss adverse effects when experiencing them or do you wait?
  - For partners: Do you think all adverse effects your partner experiences are discussed with their doctor? And what do you do when you feel that this is not the case?
  - Are there reasons not to discuss adverse effects with your treatment team (either doctor or nurse)?
  - Does the possibility of pausing or stopping the treatment influence whether or not you would discuss adverse effects with your doctor? And would receiving more information on the consequences of adverse effects influence this?
  - Do you feel free to discuss adverse effects or other symptoms with your doctor during the outpatient visit?
- Information and preferences
  - Do you see other people who receive similar treatment to exchange experiences? If so, what do you discuss with these people? If no, do you feel the need to talk to other people who receive similar treatment?
  - Do you ever search for more information regarding the treatment or regarding your diagnosis? If so, where do you find your information?
  - Is there enough information available regarding possible adverse effects you might experience while receiving your treatment?
  - For partners: Is it clear to you which adverse effects your partner might experience?
  - For partners: Do you notice it when your partner experiences adverse effects?
  - For partners: Is there any need for more information regarding adverse effects of the treatment or more information on the diagnosis? And how would you like to receive this information?
  - Are there any preferences in receiving information on adverse effects, treatment or diagnosis? For example, peer support meetings, contacts via patient organisations or Facebook groups.
- Therapeutic drug monitoring (TDM)
  - Introduction and explanation of TDM. All questions are hypothetical since TDM in TKI-related treatment is not implemented yet.
  - Would you be willing to periodically have your blood drawn to determine the TKI concentration in your blood?
  - What are your thoughts if the concentration TKI would be too high or too low?
  - What are your thoughts on TDM-based dosing of the TKI? Would your thoughts be different when talking about increase or decreasing the dose of your current TKI based on TDM?
  - What are essential elements to implement TDM in the future?
  - Would you be willing to participate in this kind of research?

**Appendix II – codes used in transcripts**

All codes used to analyse the transcripts of the focus groups. The codes are divided into groups and further specified into sub-codes.

| **Group** | **Sub-code** | **Frequency** |
| --- | --- | --- |
| Treatment-related toxicity | Bad taste | 1 |
|  | Cold | 2 |
|  | Cough | 1 |
|  | Cramps | 6 |
|  | Diarrhoea | 4 |
|  | Erysipelas | 1 |
|  | Fatigue | 7 |
|  | Fever | 2 |
|  | Flatulence | 1 |
|  | From other medication | 6 |
|  | General illness | 2 |
|  | Haemorrhage | 2 |
|  | Hair | 3 |
|  | Heart | 2 |
|  | Itching feet | 1 |
|  | Kidney | 1 |
|  | Liver | 4 |
|  | Many | 2 |
|  | Mood | 2 |
|  | Muscles | 4 |
|  | Nails | 3 |
|  | Nausea/vomiting | 3 |
|  | Neurocognitive effects | 6 |
|  | No alcohol | 1 |
|  | No appetite | 1 |
|  | None | 7 |
|  | Not recognizable | 6 |
|  | Obstipation | 2 |
|  | Oedema | 7 |
|  | Pain in feet or arms | 2 |
|  | Pain in joints | 1 |
|  | Potassium | 1 |
|  | Recognizable | 9 |
|  | Sensitivity to light and sound | 3 |
|  | Sensitivity to sun | 9 |
|  | Shaking | 2 |
|  | Skin | 7 |
|  | Transpiration | 1 |
|  | Weight gain | 3 |
| Consultation | Bad communication between hospitals | 2 |
|  | Blood results | 5 |
|  | Confronting | 3 |
|  | Defined | 1 |
|  | Difficult | 2 |
|  | Discontent | 6 |
|  | Discussing symptoms | 10 |
|  | Discussing TKI dose | 1 |
|  | Gives confidence | 5 |
|  | Insecure | 7 |
|  | No discussion of symptoms | 4 |
|  | No extra consultation | 1 |
|  | No need for extra discussion | 1 |
|  | No pain | 3 |
|  | No stress | 2 |
|  | Not defined | 1 |
|  | Often | 2 |
|  | Results physician | 8 |
|  | Results via telephone | 2 |
|  | Self-proactive | 12 |
|  | Stress | 13 |
|  | Taboo | 1 |
| Contact physician | Calling back | 1 |
|  | Content | 17 |
|  | Difficult | 2 |
|  | Discontent | 4 |
|  | General practitioner | 3 |
|  | Incomplete information | 1 |
|  | No limitations | 10 |
|  | Not calling back | 2 |
|  | Not proactive | 2 |
|  | Proactive | 5 |
|  | Relation | 7 |
|  | Waiting for consult | 1 |
| Contact nurse | Discontent | 2 |
|  | Fine | 2 |
|  | General | 4 |
|  | Not useful | 1 |
| Check-up | All in one day | 4 |
|  | Blood results | 10 |
|  | ECG | 4 |
|  | General | 2 |
|  | Good news | 3 |
|  | Not all in one day | 4 |
|  | Often | 2 |
|  | Scan | 19 |
|  | Sooner with symptoms | 1 |
|  | Tedious | 2 |
| Coping toxicities | Changes | 1 |
|  | Comments other people | 1 |
|  | Consultation with physician | 9 |
|  | Denial | 1 |
|  | Diet | 2 |
|  | Doable | 7 |
|  | Extra clothing | 1 |
|  | Extra medication | 2 |
|  | High impact | 1 |
|  | Improved | 3 |
|  | In bed a lot | 1 |
|  | Influence previous treatment | 6 |
|  | Moisturize | 1 |
|  | No complainer | 1 |
|  | None | 1 |
|  | Not discussing | 3 |
|  | Other medication | 5 |
|  | Part of it | 20 |
|  | Quality of life | 2 |
|  | Reduction | 5 |
|  | Referral | 3 |
|  | Relatively | 2 |
|  | Sensitivity to sun | 10 |
|  | Sometimes | 1 |
|  | Source unclear | 21 |
|  | Take it in to account | 9 |
|  | Waiting | 3 |
|  | Working less | 1 |
|  | Write down | 1 |
| Coping cancer | Acceptation process | 3 |
|  | Active life | 9 |
|  | Always on their mind | 4 |
|  | Always patient | 1 |
|  | Communication relatives | 4 |
|  | Conscious living | 3 |
|  | Content | 6 |
|  | Continuing daily life | 12 |
|  | Coping changes over time | 8 |
|  | Discrepancy partner and patient | 1 |
|  | Environment | 3 |
|  | Feeling good | 3 |
|  | Going for it | 3 |
|  | Impact | 11 |
|  | Incomprehension environment | 2 |
|  | Insecure | 9 |
|  | No choice | 4 |
|  | No patient | 8 |
|  | Other factors | 1 |
|  | Positive towards life | 8 |
|  | Psychologist | 1 |
|  | Stable | 4 |
| Demographic | General | 25 |
| Diagnosis | General | 4 |
|  | ALK | 13 |
|  | Biopsy | 1 |
|  | EGFR | 1 |
|  | Disbelief | 3 |
|  | Discovery | 13 |
|  | Other | 3 |
| Dose | General | 3 |
| Dose adjustment | Denied | 2 |
|  | Discussion | 1 |
|  | Reduction | 5 |
|  | Temporarily stop with reduction | 3 |
|  | Temporarily stop without reduction | 1 |
| Information | Audio recording | 1 |
|  | Difficult to understand | 2 |
|  | Facebook | 3 |
|  | Future | 2 |
|  | Information leaflet | 8 |
|  | Little is known | 2 |
|  | Medical background | 8 |
|  | None | 2 |
|  | Not searching information | 8 |
|  | Nurse | 4 |
|  | Package leaflet | 4 |
|  | Pharmaceutical company | 1 |
|  | Physician | 14 |
|  | Science | 5 |
|  | Source unclear | 1 |
|  | Website - general | 14 |
|  | Website - patient organisation | 6 |
| Peers | Able to handle it by themselves | 2 |
|  | Confronting | 5 |
|  | Dose reduction | 1 |
|  | Facebook | 1 |
|  | Forum | 2 |
|  | Gathering | 3 |
|  | General | 2 |
|  | Gives hope | 5 |
|  | Gives support | 3 |
|  | Information | 1 |
|  | Insecure | 4 |
|  | Little | 3 |
|  | Little information | 2 |
|  | No | 4 |
|  | No need to | 8 |
|  | No recognition | 7 |
|  | No time | 2 |
|  | Pleasant | 4 |
|  | Privacy | 2 |
|  | Recognition | 5 |
|  | Research | 1 |
|  | Seen | 6 |
|  | Spontaneous | 5 |
|  | Symptoms seen | 1 |
|  | Through research | 1 |
|  | Too much information | 1 |
|  | TV | 1 |
|  | Unpleasant | 5 |
|  | Walk-in-house | 2 |
| Medication | Alectinib | 13 |
|  | Brigatinib | 1 |
|  | Crizotinib | 8 |
|  | Dabrafenib and trametinib | 2 |
|  | Dose | 2 |
|  | Feeling good | 11 |
|  | Lorlatinib | 1 |
|  | Osimertinib | 1 |
|  | Other | 8 |
|  | Previous treatment | 1 |
|  | Start | 9 |
| Research | Participation | 3 |
|  | TDM | 3 |
| Partner | Acceptation process | 1 |
|  | Communication | 8 |
|  | Discontent | 1 |
|  | Friction | 1 |
|  | Insecure | 6 |
|  | Medical background | 1 |
|  | Notices little symptoms | 1 |
|  | Notices symptoms | 1 |
|  | Optimistic | 1 |
|  | Questions for physician | 5 |
|  | Sees symptoms | 7 |
|  | Stress | 4 |
|  | Support | 5 |
| Therapeutic drug monitoring | Treatment related toxicities | 5 |
|  | Contact physician important | 4 |
|  | Dose reduction | 10 |
|  | Effect on body | 9 |
|  | Finance | 3 |
|  | No pion | 6 |
|  | Frequent check-ups | 7 |
|  | Participating in research | 5 |
|  | No change as long as all is going well | 16 |
|  | Personally tailored | 8 |
|  | Trepidation | 12 |
|  | Doubts | 3 |
|  | Increase | 1 |
|  | Reduction | 6 |
|  | Efficacy guaranteed | 6 |
| Future | Hopeful | 5 |
|  | Insecure | 19 |
|  | Follow-up treatments | 12 |
|  | As long as possible TKI | 6 |
| Metastasis | General | 4 |
|  | Radiation therapy | 5 |
|  | Bone | 2 |
|  | Brain | 10 |
|  | Lung | 1 |
|  | Lymph nodes | 2 |
|  | Medication | 8 |
|  | Surgery | 1 |
| Medical history | General | 1 |
|  | Radiation therapy | 8 |
|  | Breast cancer | 1 |
|  | Bronchoscopy | 1 |
|  | Chemo therapy | 10 |
|  | CVA | 4 |
|  | Cardiac problems | 1 |
|  | Liver cancer | 1 |
|  | Lung embolism | 1 |
|  | Lung surgery | 3 |
|  | Partner with cancer | 3 |

**Appendix III – COREQ guideline checklist**

| **Topic** | **Item No.** | **Guide Questions/Description** | **Reported on**  **Page No.** |
| --- | --- | --- | --- |
| **Domain 1: Research team**  **and reﬂexivity** | | | |
| *Personal characteristics* | | | |
| Interviewer/facilitator | 1 | Which author/s conducted the interview or focus group? | 5 |
| Credentials | 2 | What were the researcher’s credentials? E.g. PhD, MD | 5 |
| Occupation | 3 | What was their occupation at the time of the study? | 5 |
| Gender | 4 | Was the researcher male or female? |  |
|  |  |  | 5 |
| Experience and training | 5 | What experience or training did the researcher have? | 5 |
| *Relationship with*  *participants* | | | |
| Relationship established | 6 | Was a relationship established prior to study commencement? | 5 |
| Participant knowledge of  the interviewer | 7 | What did the participants know about the researcher? e.g. personal  goals, reasons for doing the research |  |
|  |  |  | 5 |
|  |  |  |  |
| Interviewer characteristics | 8 | What characteristics were reported about the inter viewer/facilitator?  e.g. Bias, assumptions, reasons and interests in the research topic |  |
|  |  |  | NA |
|  |  |  |  |
| **Domain 2: Study design** | | | |
| *Theoretical framework* | | | |
| Methodological orientation and Theory | 9 | What methodological orientation was stated to underpin the study? e.g. grounded theory, discourse analysis, ethnography, phenomenology,  content analysis |  |
|  |  |  | 3/4 |
|  |  |  |  |
| *Participant selection* | | | |
| Sampling | 10 | How were participants selected? e.g. purposive, convenience,  consecutive, snowball |  |
|  |  |  | 5 |
|  |  |  |  |
| Method of approach | 11 | How were participants approached? e.g. face-to-face, telephone, mail,  email |  |
|  |  |  | 5 |
|  |  |  |  |
| Sample size | 12 | How many participants were in the study? | 7 |
| Non-participation | 13 | How many people refused to participate or dropped out? Reasons? | 7 |
| *Setting* | | | |
| Setting of data collection | 14 | Where was the data collected? e.g. home, clinic, workplace | 5 |
| Presence of non-  participants | 15 | Was anyone else present besides the participants and researchers? |  |
|  |  |  | NA |
|  |  |  |  |
| Description of sample | 16 | What are the important characteristics of the sample? e.g. demographic  data, date |  |
|  |  |  | 7/Table 1 |
|  |  |  |  |
| *Data collection* | | | |
| Interview guide | 17 | Were questions, prompts, guides provided by the authors? Was it pilot  tested? | Appendix I |
|  |  |  |  |
| Repeat interviews | 18 | Were repeat inter views carried out? If yes, how many? | NA |
| Audio/visual recording | 19 | Did the research use audio or visual recording to collect the data? | 6 |
| Field notes | 20 | Were ﬁeld notes made during and/or after the interview or focus group? | 5/6 |
| Duration | 21 | What was the duration of the inter views or focus group? | 5 |
| Data saturation | 22 | Was data saturation discussed? | 6 |
| Transcripts returned | 23 | Were transcripts returned to participants for comment and/or | NA |

| **Topic** | **Item No.** | **Guide Questions/Description** | **Reported on**  **Page No.** |
| --- | --- | --- | --- |
|  |  | correction? |  |
| **Domain 3: analysis and**  **ﬁndings** | | | |
| *Data analysis* | | | |
| Number of data coders | 24 | How many data coders coded the data? | 6 |
| Description of the coding  tree | 25 | Did authors provide a description of the coding tree? |  |
|  |  |  | Appendix II |
|  |  |  |  |
| Derivation of themes | 26 | Were themes identiﬁed in advance or derived from the data? | 6 |
| Software | 27 | What software, if applicable, was used to manage the data? | 6 |
| Participant checking | 28 | Did participants provide feedback on the ﬁndings? | NA |
| *Reporting* | | | |
| Quotations presented | 29 | Were participant quotations presented to illustrate the themes/ﬁndings?  Was each quotation identiﬁed? e.g. participant number |  |
|  |  |  | 7-11 |
|  |  |  |  |
| Data and ﬁndings consistent | 30 | Was there consistency between the data presented and the ﬁndings? | 7-11 |
| Clarity of major themes | 31 | Were major themes clearly presented in the ﬁndings? | 7-11 |
| Clarity of minor themes | 32 | Is there a description of diverse cases or discussion of minor themes? | 7-11 |

Developed from: Tong A, Sainsbury P, Craig J. Consolidated criteria for reporting qualitative research (COREQ): a 32-item checklist for interviews and focus groups. *International Journal for Quality in Health Care*. 2007. Volume 19, Number 6: pp. 349 – 357
